# Genome Sequence of a Typical Ultramicrobacterium, *Curvibacter* sp. Strain PAE-UM, Capable of Phthalate Ester Degradation

**DOI:** 10.1128/genomeA.01510-15

**Published:** 2016-01-14

**Authors:** Dan Ma, Zhenyu Hao, Rui Sun, Mark Bartlam, Yingying Wang

**Affiliations:** a Key Laboratory of Pollution Processes and Environmental Criteria (Ministry of Education), Tianjin Key Laboratory of Environmental Remediation and Pollution Control, College of Environmental Science and Engineering, Nankai University, Tianjin, China; b State Key Laboratory of Medicinal Chemical Biology & College of Life Sciences, Nankai University, Tianjin, China

## Abstract

*Curvibacter* sp. strain PAE-UM, isolated from river sediment, is a typical ultramicrobacterium capable of phthalate ester degradation. The genome of *Curvibacter* sp. PAE-UM consists of 3,284,473 bp, and its information will provide insights into the molecular mechanisms underlying its degradation ability.

## GENOME ANNOUNCEMENT

Phthalate esters (PAEs) are widely used in the manufacture of household products, pharmaceuticals, etc. as plasticizers. However, PAEs constitute one of the most frequently detected persistent organic pollutants in the environment (1). Known as endocrine-disrupting chemicals, PAEs and their degradation intermediates are reported to pose potential risks to human health (2). Microbial degradation of PAEs has attracted considerable attention, and a number of bacteria capable of degrading PAEs have been reported (1, 3, 4). However, the degradation of PAEs under oligotrophic conditions (i.e., low-nutrient concentration) has largely been neglected. *Curvibacter* sp. strain PAE-UM is the first described ultramicrobacterium capable of utilizing various types of PAEs as its sole carbon and energy source under oligotrophic conditions.

Ultramicrobacteria are defined as bacteria with a cell volume of <0.1 µm^3^, and they retain this volume irrespective of growth conditions (5). The significance of such small bacteria in the biological cycling of nutrients has been recognized by microbial ecologists (6, 7). *Curvibacter* sp. PAE-UM is a typical ultramicrobacterium possessing a distinctive small biovolume (<0.05 µm³). It was isolated from sediment in the Songhua River using the extinction dilution method in combination with flow cytometric analysis (8). It was cultured under oligotrophic conditions with sterilized mineral water as the medium (7) and PAEs as the sole carbon and energy source. Strain PAE-UM can mineralize different PAEs, including dimethyl phthalate, diethyl phthalate, di-*n*-butyl phthalate, di(2-ethylhexyl) phthalate, and butyl benzyl phthalate.

The complete genome of *Curvibacter* sp. strain PAE-UM was sequenced using the Illumina HiSeq 2000 sequencing platform. Genomic libraries were constructed containing 500-bp paired-end (PE) and 6-kbp mate-pair (MP) inserts to generate hybrid 906 Mb of data, assembled into 3,632,663 bp and providing 249.4-fold coverage of the genome. The reads, assembled into 36 contigs utilizing *de novo* assembly, were used to construct 4 scaffolds. The genome was submitted to the NCBI Prokaryotic Genome Annotation Pipeline (PGAP) for annotation. The functions of predicted protein-coding genes were annotated through comparisons with the NCBI nonredundant (NR) and Swiss-Prot databases from the European Bioinformatics Institute.

The genome has a total length of 3,632,663 bp, with a G+C content of 65.5%. Further analysis showed that the genome contains 3,409 genes, which make up 90.4% of the whole genome. According to tRNAscan-SE (9) and RNAmmer 1.2 (10), there are 3 rRNA operons and 48 tRNA genes in the genome. Strain PAE-UM belongs to the genus *Curvibacter* on the basis of 16S rRNA gene sequencing and genetic comparison with the strain *Curvibacter* sp. R-36930 (accession no. NC_ FR691424). Functional annotation showed that strain PAE-UM possesses a number of PAE-degrading genes, including genes encoding for esterase, 4,5-dihydroxyphthalate decarboxylase, protocatechuate 3,4-dioxygenase, 2-pyrone-4,6-dicarboxylate-degrading genes, 4-oxalomesaconate hydratase, 4-carboxy-4-hydroxy-2-oxoadipate aldolase, and others.

In summary, we have sequenced the complete genome of a typical ultramicrobacterium, *Curvibacter* sp. strain PAE-UM, capable of degrading PAEs under oligotrophic conditions. The complete genome sequence of strain PAE-UM will contribute toward a greater understanding of the molecular mechanisms underlying PAE degradation and provide new perspectives on the ecological functions of ultramicrobacteria.

### Nucleotide sequence accession number.

The complete genome sequence of *Curvibacter* sp. strain PAE-UM has been deposited in GenBank under the accession number LKCX00000000.

## References

[1] Net S, Sempéré R, Delmont A, Paluselli A, Ouddane B 2015 Occurrence, fate, behavior and ecotoxicological state of phthalates in different environmental matrices. Environ Sci Technol 49:4019–4035. doi:10.1021/es505233b. 25730609

[2] Bui TT, Giovanoulis G, Cousins AP, Magnér J, Cousins IT, de Wit CA 2016 Human exposure, hazard and risk of alternative plasticizers to phthalate esters. Sci Total Environ 541:451–467. doi:10.1016/j.scitotenv.2015.09.036. 26410720

[3] Jin L, Sun X, Zhang X, Guo Y, Shi H 2014 Co-metabolic biodegradation of DBP by *Paenibacillus* sp. S-3 and H-2. Curr Microbiol 68:708–716. doi:10.1007/s00284-014-0533-8. 24504631

[4] Zhang X-Y, Fan X, Qiu Y-J, Li C-Y, Xing S, Zheng Y-T, Xu J-H 2014 Newly identified thermostable esterase from *Sulfobacillus acidophilus*: properties and performance in phthalate ester degradation. Appl Environ Microbiol 80:6870–6878. doi:10.1128/AEM.02072-14. 25149523 PMC4249001

[5] Schut F, Prins R, Gottschal J 1997 Oligotrophy and pelagic marine bacteria: facts and fiction. Aquat Microb Ecol 12:177–202. doi:10.3354/ame012177.

[6] Duda VI, Suzina NE, Polivtseva VN, Boronin AM 2012 Ultramicrobacteria: formation of the concept and contribution of ultramicrobacteria to biology. Microbiology 81:379–390. doi:10.1134/S0026261712040054. 23156684

[7] Wang Y, Hammes F, Boon N, Chami M, Egli T 2009 Isolation and characterization of low nucleic acid (LNA)-content bacteria. ISME J 3:889–902. doi:10.1038/ismej.2009.46. 19421234

[8] Wang Y, Hammes F, De Roy K, Verstraete W, Boon N 2010 Past, present and future applications of flow cytometry in aquatic microbiology. Trends Biotechnol 28:416–424. doi:10.1016/j.tibtech.2010.04.006. 20541271

[9] Lowe TM, Eddy SR 1997 tRNAscan-SE: a program for improved detection of transfer RNA genes in genomic sequence. Nucleic Acids Res 25:955–964. doi:10.1093/nar/25.5.0955. 9023104 PMC146525

[10] Lagesen K, Hallin P, Rødland EA, Staerfeldt H-H, Rognes T, Ussery DW 2007 RNAmmer: consistent and rapid annotation of ribosomal RNA genes. Nucleic Acids Res 35:3100–3108. doi:10.1093/nar/gkm160. 17452365 PMC1888812

